# A facile in vitro platform to study cancer cell dormancy under hypoxic microenvironments using CoCl_2_

**DOI:** 10.1186/s13036-018-0106-7

**Published:** 2018-08-03

**Authors:** Hak Rae Lee, Faith Leslie, Samira M. Azarin

**Affiliations:** Department of Chemical Engineering and Materials Science, University of Minnesota, Minneapolis, MN 55455 USA

**Keywords:** Cancer dormancy, Hypoxia, Cobalt chloride, In vitro model, Tumor microenvironment, Tumor recurrence

## Abstract

**Background:**

While hypoxia has been well-studied in various tumor microenvironments, its role in cancer cell dormancy is poorly understood, in part due to a lack of well-established in vitro and in vivo models. Hypoxic conditions under conventional hypoxia chambers are relatively unstable and cannot be maintained during characterization outside the chamber since normoxic response is quickly established. To address this challenge, we report a robust in vitro cancer dormancy model under a hypoxia-mimicking microenvironment using cobalt chloride (CoCl_2_), a hypoxia-mimetic agent, which stabilizes hypoxia inducible factor 1-alpha (HIF1α), a major regulator of hypoxia signaling.

**Methods:**

We compared cellular responses to CoCl_2_ and true hypoxia (0.1% O_2_) in different breast cancer cell lines (MCF-7 and MDA-MB-231) to investigate whether hypoxic regulation of breast cancer dormancy could be mimicked by CoCl_2_. To this end, expression levels of hypoxia markers HIF1α and GLUT1 and proliferation marker Ki67, cell growth, cell cycle distribution, and protein and gene expression were evaluated under both CoCl_2_ and true hypoxia. To further validate our platform, the ovarian cancer cell line OVCAR-3 was also tested.

**Results:**

Our results demonstrate that CoCl_2_ can mimic hypoxic regulation of cancer dormancy in MCF-7 and MDA-MB-231 breast cancer cell lines, recapitulating the differential responses of these cell lines to true hypoxia in 2D and 3D. Moreover, distinct gene expression profiles in MCF-7 and MDA-MB-231 cells under CoCl_2_ treatment suggest that key cell cycle components are differentially regulated by the same hypoxic stress. In addition, the induction of dormancy in MCF-7 cells under CoCl_2_ treatment is HIF1α-dependent, as evidenced by the inability of HIF1α-suppressed MCF-7 cells to exhibit dormant behavior upon CoCl_2_ treatment. Furthermore, CoCl_2_ also induces and stably maintains dormancy in OVCAR-3 ovarian cancer cells.

**Conclusions:**

These results demonstrate that this CoCl_2_-based model could provide a widely applicable in vitro platform for understanding induction of cancer cell dormancy under hypoxic stress.

**Electronic supplementary material:**

The online version of this article (10.1186/s13036-018-0106-7) contains supplementary material, which is available to authorized users.

## Background

During tumor progression, some disseminated tumor cells (DTCs) are capable of surviving in a prolonged quiescent state [1]. These dormant cells can reside at secondary sites without any clinical evidence for months to years before reawakening and causing metastatic recurrence [2, 3], as evidenced by the large fraction of cancer patients who exhibit an asymptomatic period before metastatic relapse [1, 4]. Developing strategies to destroy these dormant DTCs will require a better understanding of their unique biological characteristics, as they are able to evade current chemotherapeutic approaches that target rapidly dividing cells. It has been postulated that DTC dormancy could be induced by microenvironmental stresses encountered by the cells either within the primary tumor prior to dissemination or upon arrival at a secondary site [1, 5]. Over the past few decades, significant effort has been directed toward understanding how microenvironmental cues regulate cancer dormancy [3, 6, 7]. However, the lack of suitable platforms to induce and maintain dormancy has limited the ability to probe the switch between dormant and active states in cancer cells.

Accumulating evidence in multiple types of cancer has revealed that dormant DTCs are detected in the bone marrow at a particularly high rate, suggesting that DTCs favor bone marrow despite its hostile microenvironmental conditions such as hypoxia and hypoglycemia [3, 8]. Previous analysis of breast cancer cells has identified several genes involved in dormancy that are regulated by hypoxia [9, 10]. In addition, a recent study found that hypoxic microenvironments in the primary tumor give rise to a subpopulation of DTCs programmed to become dormant [11]. However, the precise role of hypoxia in regulating dormancy remains poorly understood due to a lack of well-established in vivo and in vitro models. Hypoxia studies are typically performed using incubation chambers that maintain an oxygen depleted environment through regulation of gas composition in the chamber [12, 13]. These chambers limit the range of conditions that can be evaluated in an individual study, and the cells quickly establish normoxia each time they are removed for manipulation. In vivo models have the advantage of recapitulating the complex microenvironment of the tumor or metastatic site, but dormant cells are rare and thus it is difficult to identify and isolate them in vivo*.* In addition, regulation of hypoxia in vivo requires placement of mice in hypoxia chambers, which limits study size and also tunability of the hypoxic environment. In vitro models also present challenges, as the cells must be maintained in both hypoxic and dormant states, both of which are relatively unstable, during characterization. Thus, we sought to develop a robust in vitro model capable of stably inducing and maintaining dormancy of cancer cells under hypoxic microenvironments.

In this work, CoCl_2_, a well-known hypoxia-mimetic agent, was used to establish hypoxia-mimicking microenvironments in vitro. The response to hypoxia is generally characterized by expression of the heterodimeric hypoxia induction factor 1 (HIF1) protein that consists of two subunits: HIF1α and HIF1β. HIF1β is constitutively expressed in the nucleus, whereas HIF1α is regulated by oxygen tension. It has been shown that the HIF-specific prolyl hydroxylases that facilitate HIF1α degradation have an iron-binding core, and the iron at this core is thought to be essential for their enzymatic activities [14]. This iron can be replaced by cobalt, resulting in the inhibition of HIF1α degradation [14]. In addition, cobalt inhibits the interaction between HIF1α and von Hippel Lindau (VHL) protein, another protein involved in HIFα degradation, thereby preventing the degradation of HIF1α [15]. Since CoCl_2_ mimics hypoxia by stabilizing HIF1α expression regardless of oxygen levels, this method has the advantage of being more stable than conventional hypoxic chambers. In addition, it has been demonstrated that CoCl_2_ and true hypoxia result in similar regulation of hypoxia-related downstream targets such as erythropoietin and glucose transporter 1 (GLUT1) [16–18]. It has been documented that CoCl_2_ can be used to mimic hypoxia in multiple cancer cell lines including breast and ovarian cancer cells [19, 20]. While the ability of CoCl_2_ to mimic hypoxic conditions in cancer cells has been established, it has not yet been demonstrated that the induction of dormancy in cancer cells lines in response to hypoxia can be recapitulated by CoCl_2_.

In this manuscript, we evaluate whether CoCl_2_-induced hypoxia-mimicking microenvironments can trigger and maintain dormancy in vitro in breast and ovarian cancer cell lines, with cancer dormancy defined as reversible quiescence throughout this report. Moreover, we show that CoCl_2_ affects tumor dormancy directly through HIF1α stabilization by investigating effects of CoCl_2_ on MCF-7 cells containing knockdown of HIF1α expression. In addition, we investigate whether the cellular response to CoCl_2_ recapitulates the differential response to true hypoxia in estrogen receptor (ER)-positive and ER-negative breast cancer cells, which exhibit different dormancy signatures in vivo [9], to further validate our model. This CoCl_2_-based model offers a facile tool for detailed investigation of cancer dormancy under hypoxic conditions, which could serve as an enabling platform to further understanding of how the dormant state is regulated in cancer cells.

## Methods

### Cell culture and growth analysis

The human breast cancer cell lines, MCF-7 and MDA-MB-231 (ATCC), were maintained in Dulbecco’s Modified Eagle’s Media (DMEM, 4500 mg/L glucose, Sigma Aldrich) supplemented with 10% (*v*/v) fetal bovine serum (FBS; Thermo Fisher Scientific) and 1% (v/v) penicillin-streptomycin (PS; Thermo Fisher Scientific). The human ovarian cancer cell line, OVCAR-3 (ATCC), was cultured in Roswell Park Memorial Institute 1640 (RPMI 1640) media supplemented with 10% (*v*/v) FBS, 1% (v/v) PS, and 0.001% (*w*/*v*) bovine insulin (Sigma Aldrich). For cell growth analysis, cells were seeded at a density of 1 × 10^5^ cells per well in 6-well plates or 35 mm dishes. Prior to counting, cells were singularized using 0.25% trypsin-EDTA (Thermo Fisher Scientific) and treated with a 1:1 ratio of Trypan blue (Thermo Fisher Scientific), after which the live cell number was determined using a Countess II FL automated cell counter (Thermo Fisher Scientific).

### Generation of cell aggregates and encapsulation of cells for three-dimensional (3D) models

Cell aggregates were generated using non-adhesive poly(2-hydroxyethyl methacrylate; pHEMA, Sigma Aldrich)-coated plates. pHEMA was dissolved in 95% ethanol to a final concentration of 3 mg/ml and sterile-filtered. The pHEMA solution was added to cell culture plates, which were left to dry overnight at room temperature. Cells were seeded in the pHEMA-coated plates at a density of 5 × 10^4^ cells/cm^2^ for aggregate generation. Collagen gels were prepared by diluting collagen type I (rat tail, Corning) with cell culture media to a final concentration of 2.5 mg/ml and neutralizing with 1 N sodium hydroxide (Sigma Aldrich). Cells were mixed with the diluted collagen solution at a density of 5 × 10^6^ cells/ml, and 100 μl of the solution was plated in 35 mm dishes and gelled at 37 °C for 30 min. In order to harvest cells for characterization, cell aggregates were singularized by StemPro Accutase (Gibco) for 15 min with gentle pipetting, and collagen gels were dissociated by collagenase type 1 (Sigma Aldrich) for 15 min at 37 °C.

### CoCl_2_ treatment and exposure to true hypoxia

For 2D cultures, treatment with CoCl_2_ or true hypoxia was initiated when cells reached 50% confluence. For 3D cultures, treatment with CoCl_2_ or true hypoxia was initiated immediately after cells were seeded in pHEMA-coated plates or embedded in collagen gels. For CoCl_2_ treatment, CoCl_2_ (Sigma Aldrich) was dissolved in distilled water and sterile-filtered. The resulting aqueous CoCl_2_ solution was directly added to the cell culture media. Hypoxic culture was performed by incubating cells with 0.1% O_2_ and 5% CO_2_ in the EVOS FL Auto on-stage incubator (Thermo Fisher Scientific).

### Cell cycle analysis

Cells cultured for the indicated time periods were singularized using 0.25% trypsin-EDTA (Thermo Fisher Scientific) and fixed with 70% ethanol for 1 h at − 20 °C. Then, cells were stained with 50 μg/mL propidium iodide (PI; Thermo Fisher Scientific) for 30 min in the dark at room temperature. Flow cytometric analysis of PI staining intensity was performed using a LSR II flow cytometer (BD Biosciences), and data were analyzed using Modfit LT software (Verity Software House) to determine cell cycle distribution.

### Immunofluorescence assays

Cells grown on glass-bottom dishes or well plates were fixed with 4% paraformaldehyde for 10 min at room temperature. Cells embedded in collagen gels or cell aggregates grown in pHEMA-coated plates were fixed for 20 min at room temperature. Blocking and permeabilization were performed in blocking buffer, phosphate buffered saline (PBS) containing 10% Normal Goat Serum (Thermo Fisher Scientific) and 0.3% Triton X-100 (Sigma Aldrich), for 1 h. Then, the cells were stained with rabbit anti-human Ki67 (D3B5; 1:400 dilution, Cell Signaling Technology), mouse anti-human HIF1α (54/HIF1α, 1:200, BD Biosciences), or mouse anti-human GLUT1 (SPM498, 1:200, Thermo Fisher Scientific) in antibody dilution buffer, PBS containing 1% bovine serum albumin (Sigma Aldrich) and 0.3% Triton X-100, overnight at 4 °C. Next, cells were labeled for 1 h at room temperature with corresponding secondary antibodies: goat anti-rabbit Alexa Fluor 594 (1:1000, Thermo Fisher Scientific) for Ki67, goat anti-mouse Alexa Fluor 647 (1:1000, Thermo Fisher Scientific) for HIF1α, and goat anti-mouse Alexa Fluor 488 (1:1000, Thermo Fisher Scientific) for GLUT1. Nuclei were labeled with DAPI (300 nM, Thermo Fisher Scientific). Samples were imaged using an EVOS FL Auto fluorescence microscope, with the same light intensity and exposure time applied across all samples. Quantification was performed with ImageJ software (National Institutes of Health).

### Western blotting

For protein extraction, cells were washed twice with ice-cold PBS and lysed using RIPA buffer (Thermo Fisher Scientific). Whole cell lysates were separated on a SDS-PAGE gradient gel (4–15%) and transferred to a polyvinylidene difluoride (PVDF) membrane (Bio-Rad). HIF1α, p21, p27, p38 mitogen-activated protein kinase (MAPK), phospho-p38 (pp38) MAPK, extracellular signal-regulated kinase (ERK(1/2)), and phospho-ERK(1/2) (pERK(1/2)) were detected in 30 μg of whole cell lysates. β-actin was detected in 10 μg of whole cell lysates. The membranes were blocked in 5% non-fat dry milk (Bio-Rad) or bovine serum albumin (Sigma Aldrich) in Tris-buffered saline containing 0.05% Tween-20 (TBS-T; Sigma Aldrich) at room temperature for 1 h. Next, the membranes were incubated with mouse anti-human HIF1α primary antibody (54/HIF1α, 1:1000, BD Biosciences) for 2 h or HRP (horseradish peroxidase)-conjugated β-actin (13E5, 1:2000, Cell Signaling Technology) for 1 h at room temperature. For p21, p27, p38 MAPK, pp38 MAPK, ERK(1/2), and pERK(1/2), the membranes were incubated with rabbit anti-human p21 primary antibody (12D1), rabbit anti-human p27 primary antibody (D69C12), rabbit anti-human p38 MAPK primary antibody, rabbit anti-human pp38 MAPK primary antibody, rabbit anti-human ERK(1/2) primary antibody and rabbit anti-human pERK(1/2) primary antibody (1:1000, Cell Signaling Technology) overnight at 4 °C. For HIF1α blots, the membrane was incubated with goat anti-mouse polyclonal HRP-conjugated secondary antibody (1:10000, Thermo Fisher Scientific) at room temperature for 45 min. For p21, p27, p38 MAPK, pp38 MAPK, ERK(1/2), and pERK(1/2), goat anti-rabbit polyclonal HRP-conjugated secondary antibody (1:2000, Cell Signaling Technology) was used. Protein expression was detected using SuperSignal West Pico Chemiluminescent substrate (Thermo Fisher Scientific) and a ChemiDoc Imager (Bio-Rad). Western blot quantification was performed by ImageLab (Bio-Rad). The p38 to ERK activity ratio was calculated as previously reported [21]. Briefly, quantified values for pp38 MAPK were divided by the values for total p38 MAPK and the same was done for pERK(1/2) and ERK(1/2). Then, the pp38 MAPK/p38 MAPK ratio value was divided by pERK(1/2)/ERK(1/2) ratio value.

### Cell viability assays

The cell viability under CoCl_2_ treatment was measured using a Live/Dead Viability/Cytotoxicity Kit (Thermo Fisher Scientific). For this assay, 1 × 10^5^ cells per well were seeded in 6-well plates. After the indicated periods of CoCl_2_ treatment, cells were washed with PBS twice and incubated with 2 μM calcein AM and 4 μM ethidium homodimer-1 for 30 min at room temperature. Imaging was performed using an EVOS FL Auto fluorescence microscope.

### HIF1α knockdown in MCF-7 cells

For HIF1α suppression in MCF-7 cells, a HIF1α-specific shRNA construct in a lentiviral GFP vector was used (5’ ACAAGAACCTACTGCTAATGCCACCACTA 3′, OriGene, TL320380D). A non-effective scrambled shRNA cassette (5’ GCACTACCAGAGCTAACTCAGATAGTACT 3′, OriGene, TR30021) was used as a negative control. To produce lentiviral particles, human embryonic kidney 293 T cells (HEK293T; kindly provided by Dr. Benjamin Hackel) were transfected with HIF1α-specific shRNA constructs and the Lenti-Vpak packaging kit (OriGene, TR30037) in Opti-MEM I (Thermo Fisher Scientific). Lentiviral particles were collected 24 h after transfection and filtered through a 0.45 μm syringe filter (Merck Millipore). For transduction, MCF-7 cells were transduced with the lentiviral particles for 24 h in DMEM containing 10% FBS and polybrene (8 μg/mL, Sigma Aldrich). Selection of successfully transduced cells was achieved by exposing the cells to DMEM containing 10% FBS and 0.5 μg/mL puromycin (Life Technologies) for one week, with media changes performed every 2–3 days.

### Quantitative real-time PCR (qRT-PCR)

qRT-PCR was performed using a CFX Connect Real-Time PCR Detection System (Bio-Rad). Total RNA was extracted from cells using a RNeasy Mini kit (Qiagen) followed by cDNA synthesis through the reverse transcription of 1 μg of total RNA using an Omniscript RT kit (Qiagen) according to the manufacturer’s protocol. Quantitative PCR reactions were performed using iTaq Universal SYBR Green Supermix (Bio-Rad) and PrimePCR SYBR Green Assays (Bio-Rad) with primers specific to each of the target mRNAs: CDKN1A (qHsaCID0014498), CDKN1B (qHsaCID0012509), CDK2 (qHsaCED0043984), CDK4 (qHsaCED0003626), CCNA2 (qHsaCID0017452), CCND1 (qHsaCID0013833), CCNE1 (qHsaCID0015131), and MYC (qHsaCID0012921). TBP (TATA-box binding protein, qHsaCID0007122) was used as reference gene.

### Senescence-associated β-galactosidase assay

β-galactosidase activity was measured using the Senescence β-Galactosidase Staining Kit (Cell Signaling Technology). Briefly, cells were washed with PBS and fixed with 1× fixative solution for 15 min. Then, β-galactosidase staining solution with a final pH between 5.9 and 6.1 was prepared and added to fixed cells. Samples were sealed with parafilm to prevent evaporation and placed in a dry 37 °C incubator overnight. Imaging was performed using an EVOS FL Auto fluorescence microscope. For the positive control, cells were treated with 12.5 μM etoposide (Cell Signaling Technology) for 6 days and allowed to recover for 2 days in normal growth media.

### Statistical analysis

All data are represented as mean ± SD of three biological replicates from one of three representative independent experiments. *P* values were determined using an unpaired Student’s t-test, with *P* < 0.05 considered to be statistically significant. Statistical analysis was performed using GraphPad Prism.

## Results

### CoCl_2_ treatment inhibits proliferation of MCF-7 cells

**Fig. 1:**
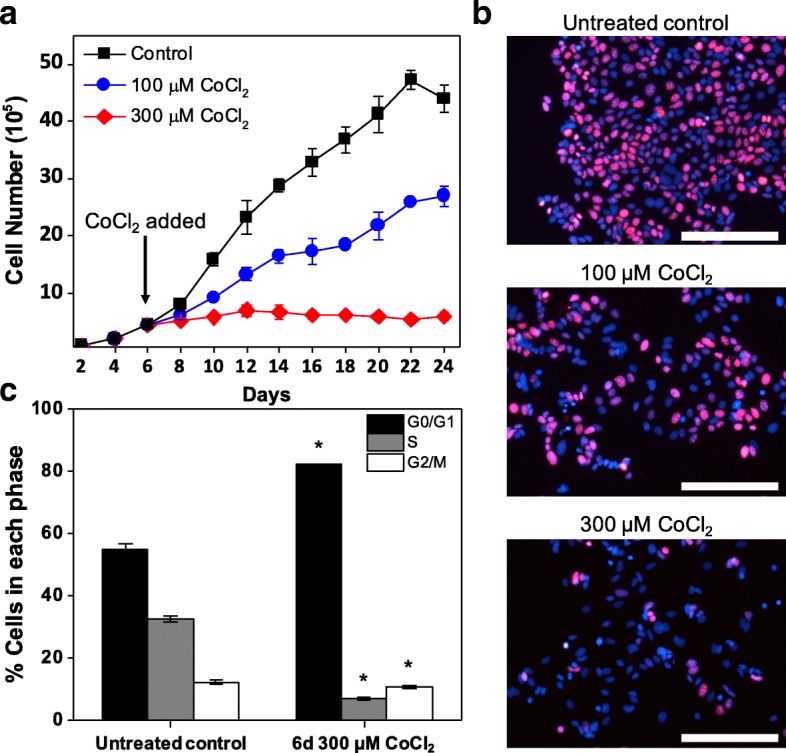
CoCl_2_-induced hypoxia-mimicking conditions can lead to prolonged growth inhibition in MCF-7 cells. Cell growth analysis (**a**) and representative fluorescence images of cell cycling marker Ki67 (red) expression (**b**) in MCF-7 cells treated with 100 and 300 μM CoCl_2_ compared to untreated control cells (day 6 of culture). Immunofluorescence analysis of Ki67 was performed after 6 days of CoCl_2_ treatment. Nuclei were stained with DAPI. Scale bars indicate 200 μm. **c** Flow cytometric analysis of MCF-7 cell cycle distribution through propidium iodide (PI) staining intensity in cells treated with 300 μM CoCl_2_ for 6 days compared to untreated cells (day 6 of culture). Data were analyzed by Modfit LT software (^*^ *P* < 0.001 compared to untreated control)

To explore the growth dynamics of MCF-7 cells under hypoxia-mimicking conditions induced by CoCl_2_ treatment, MCF-7 cells were exposed to various concentrations of CoCl_2_ (50–500 μM). Hypoxia-mimicking effects of CoCl_2_ were verified under tested concentrations through comparison to true hypoxic conditions (0.1% O_2_) by western blot analysis of HIFα (Additional file 1: Figure S1A, B) and immunofluorescence analysis of HIFα and GLUT1 (Additional file 1: Figure S1C). Inhibition of MCF-7 proliferation without significant cell death was observed in a dose-dependent manner up to 300 μM and maintained for approximately 20 days under CoCl_2_ treatment (Fig. 1a and Additional file 1: Figure S2). At doses of 1 mM or higher, toxicity led to significant cell death (Additional file 1: Figure S2), whereas doses lower than 50 μM did not result in distinguishable changes in cell growth (data not shown). Importantly, Ki67 (a cellular marker for actively cycling cells) was markedly attenuated in MCF-7 cells showing restrained growth under CoCl_2_ treatment (Fig. 1b), implying that growth inhibition is in part attributed to emergence of a quiescent population of MCF-7 cells. In addition, the attenuated Ki67 expression and restrained growth of MCF-7 became more noticeable at 300 μM CoCl_2_ than 100 μM CoCl_2_, indicating the dose-dependent cellular response of MCF-7 to CoCl_2_ treatment. Consistent with the restrained growth and attenuated Ki67 expression, during CoCl_2_ treatment a significant increase in the cell population arrested in G0/G1 phase, a hallmark of the quiescent state, was observed through flow cytometric analysis of propidium iodide (PI) staining. In MCF-7 cells treated with 300 μM CoCl_2_ for 6 days, 82.2 ± 0.1% of the cells were in G0/G1 phase, as compared to 55.0 ± 1.7% prior to CoCl_2_ treatment (Fig. 1c). In addition, the population in S phase decreased from 32.6 ± 0.9% to 7.1 ± 0.3% after 6 days of CoCl_2_ treatment (Fig. 1c). Taken together, the restrained growth in conjunction with downregulation of Ki67, actively present in G1, S, G2 and M phases of the cell cycle but absent in G0 phase [22, 23], and accumulation of cells in the G0/G1 phase by PI analysis demonstrate that at doses which increase HIF1α expression, CoCl_2_-induced hypoxia-mimicking conditions can trigger dormancy in MCF-7 cells.

### Dormant cells resume proliferation upon removal of CoCl_2_

**Fig. 2:**
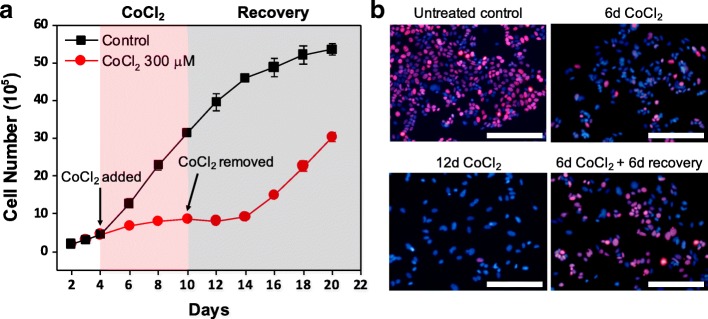
Quiescent MCF-7 cells can resume growth upon removal of CoCl_2_. **a** Cell growth analysis of MCF-7 cells treated with 300 μM CoCl_2_ for 6 days (from day 4 to day 10) compared to untreated control cells. Cells recovered in normal growth media after the 6-day CoCl_2_ treatment. **b** Representative fluorescence images of cycling marker Ki67 (red) and nuclei (blue) in MCF-7 cells after 6 days of treatment with 300 μM CoCl_2_, 12 days of treatment with 300 μM CoCl_2_, or 6 days of treatment with 300 μM CoCl_2_ followed by 6 days of recovery. The untreated control consists of cells at day 4, prior to CoCl_2_ treatment. Scale bars indicate 200 μm

Unlike senescent cells, which are permanently trapped in a non-proliferative state, a hallmark of dormant cancer cells is the ability to reawaken upon removal of the environmental stresses that led them to enter dormancy, defined as reversible quiescence in this study [24, 25]. Thus, we determined whether the cell growth inhibition under CoCl_2_ treatment could be reversed. To this end, MCF-7 cells were treated with 300 μM CoCl_2_ for 6 days (from day 4 to day 10 of cell culture) followed by recovery in the normal growth media (from day 10 to day 16, Fig. 2). Cell growth analysis showed that while proliferation was restrained during the 6-day CoCl_2_ treatment period, cells resumed growth after removal of CoCl_2_ (Fig. 2a), providing further evidence that growth-arrested cells under CoCl_2_ treatment were dormant and not senescent.

Reversible quiescence of MCF-7 cells under CoCl_2_ treatment was also observed by Ki67 immunofluorescence analysis (Fig. 2b, Additional file 1: Figure S3). The 6-day exposure to 300 μM CoCl_2_ led to significant loss of Ki67 expression in the majority of MCF-7 cells (Fig. 2b, top right) as compared to control cells prior to treatment. After 12 days of CoCl_2_ treatment without recovery, Ki67-positive cells were hardly seen, suggesting that most cells under prolonged hypoxia-mimicking conditions remained dormant (Fig. 2b, bottom left). When cells recovered from CoCl_2_ treatment for 6 days, expression of Ki67 increased, indicating that cells were re-entering a proliferative state (Fig. 2b bottom right). Flow cytometric analysis following PI staining also confirmed reversible quiescence of MCF-7 cells under CoCl_2_-induced hypoxia-mimicking conditions. After 6 days of recovery in normal growth media following CoCl_2_ treatment, the percentage of cells arrested in G0/G1 decreased from 88.0 ± 1.2% at day 10 to 53.0 ± 0.4% at day 16, while the percentage of cells in S phase increased from 6.3 ± 0.1% to 30.9 ± 0.2% (Additional file 1: Figure S4). This transient growth arrest of MCF-7 cells under CoCl_2_ treatment indicates that MCF-7 cells were in a dormant state, as opposed to a senescent state. To further demonstrate that the cells were not senescent, we evaluated senescence-associated β-galactosidase activity. MCF-7 cells treated with CoCl_2_ had low β-galactosidase activity similar to untreated cells, whereas MCF-7 cells treated with etoposide, a well-known inducer of cell senescence, exhibited higher β-galactosidase activity (Additional file 1: Figure S5). Collectively, these results confirm that MCF-7 cells under CoCl_2_-induced hypoxia-mimicking conditions are dormant and resume proliferation when CoCl_2_ is removed.

### Induction of dormancy by CoCl_2_ is similar to true hypoxia

**Fig. 3:**
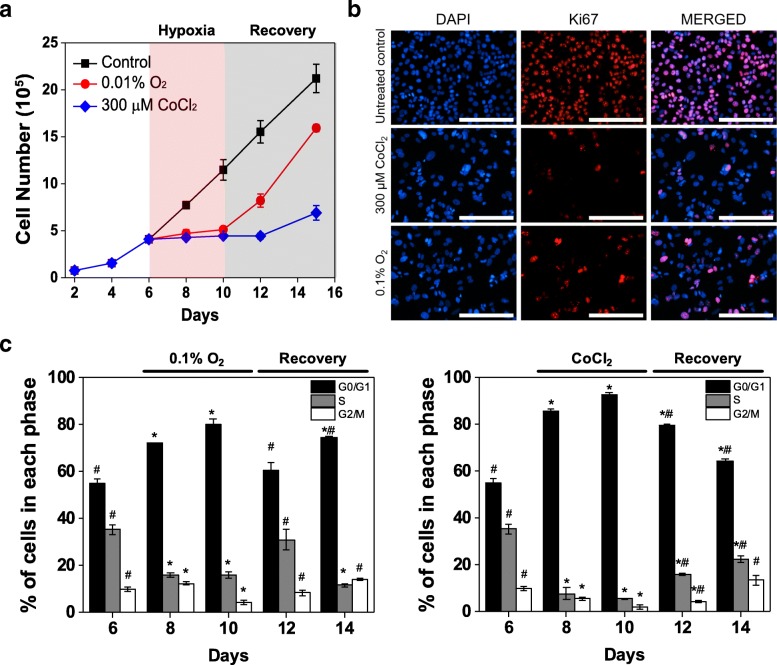
Induction of quiescence by CoCl_2_ treatment is similar to effects of true hypoxia. **a** Cell growth analysis of MCF-7 cells treated with 300 μM CoCl_2_ or true hypoxic conditions (0.1% O_2_) for 4 days (from day 6 to day 10) compared to untreated cells. **b** Representative fluorescence images of Ki67 expression in MCF-7 cells after 6 days of exposure to 300 μM CoCl_2_ or 0.1% O_2_ compared to untreated control cells (day 6 of culture). Nuclei were stained with DAPI. Scale bars indicate 200 μm. **c** Flow cytometric analysis of PI staining in cells exposed to true hypoxic conditions (0.1% O_2_, left) or 300 μM CoCl_2_ treatment (right). Cell populations are reported as percentage of cells in each phase. Data were analyzed by Modfit LT software (^*^ *P* < 0.05 compared to untreated control (day 6); ^#^ P < 0.05 compared to day 10 of the respective condition)

We subsequently compared cell responses under true hypoxic conditions and CoCl_2_ treatment to confirm that dormancy hallmarks of MCF-7 cells under CoCl_2_ and true hypoxia are similar. To this end, MCF-7 cells were exposed to 300 μM CoCl_2_ or true hypoxic conditions (0.1% O_2_). Cell growth analysis demonstrated similar inhibition of cell growth during the treatment period (Fig. 3a). During recovery, cells in both conditions resumed growth upon removal of either CoCl_2_ or true hypoxia. The short delay observed in CoCl_2_ treatment, but not in true hypoxia, could be attributed to the extended time required for elimination of residual CoCl_2_ that has visibly accumulated within the cells, delaying recovery from hypoxia-mimicking conditions even after CoCl_2_ has been removed from the culture media. Immunofluorescence analysis further supported that both conditions led to similar responses, with exposure to 300 μM CoCl_2_ and 0.1% O_2_ both leading to attenuated expression of Ki67 (Fig. 3b). Flow cytometric analysis of PI staining further demonstrated that both conditions led to a significant increase in the cell population arrested in G0/G1 phase, 80.0 ± 2.2% under true hypoxia and 92.5 ± 0.7% under CoCl_2_ treatment (day 10), compared with 55.0 ± 1.8% in untreated MCF-7 cells (day 6; Fig. 3c). During normoxic recovery, a reduction in the arrested G0/G1 cell population was observed in both conditions, with 60.7 ± 3.2% G0/G1 phase cells in the population recovering from true hypoxia at day 12 and 64.0 ± 1.2% G0/G1 phase cells in the population recovering from CoCl_2_ treatment at day 14 (Fig. 3c), indicating that cells under both conditions exhibited reversible quiescence. The increase in the G0/G1 cell population at day 14 observed in cells recovering from true hypoxia could be attributed to contact inhibition, as cells recovered and proliferated rapidly, reaching confluence in the culture dish. Collectively, these results show that the regulation of cancer dormancy under true hypoxic conditions can be mimicked by CoCl_2_.

### Effects of CoCl_2_ on cancer dormancy result directly from modulation of HIF1α

**Fig. 4:**
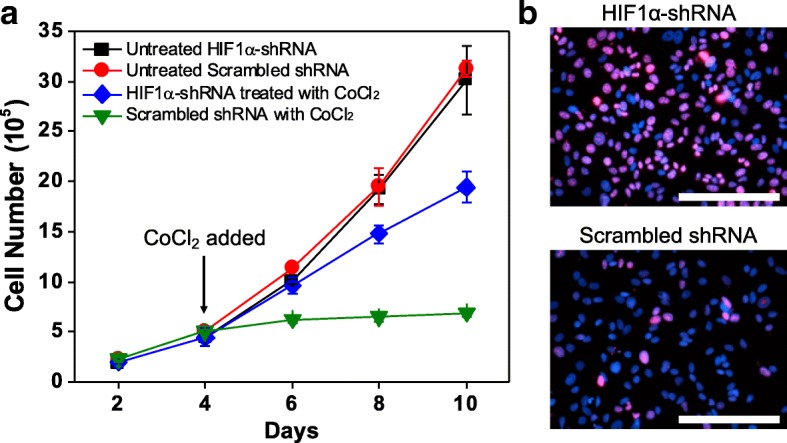
Induction of dormancy by CoCl_2_ is dependent on HIF1α. **a** Cell growth analysis of HIF1α-suppressed MCF-7 cells treated with 300 μM CoCl_2_ for 6 days (from day 4 to day 10) compared to untreated HIF1α-suppressed MCF-7 cells. Treated and untreated MCF-7 cells transduced with shRNA containing a scrambled sequence served as additional control groups. **b** Representative fluorescence images of Ki67 (red) and nuclei (blue) in HIF1α-suppressed MCF-7 cells (top) and MCF-7 cells transduced with scrambled shRNA (bottom) after 4-day treatment with 300 μM CoCl_2_. Scale bars indicate 200 μm

To verify that unknown effects of CoCl_2_, separate from HIF1α-stabilizing effects, were not responsible for induction of cancer dormancy, we tested whether CoCl_2_ could induce dormancy in breast cancer cells in a HIF1α-independent manner. To this end, we knocked down HIF1α expression in MCF-7 cells using short hairpin RNA (shRNA). MCF-7 cells transduced with HIF1α-specific shRNA demonstrated significantly suppressed expression of HIF1α upon treatment with 300 μM CoCl_2_, as compared to MCF-7 cells transduced with shRNA containing a scrambled sequence (Additional file 1: Figure S1B and Figure S6). Furthermore, the growth of HIF1α-suppressed MCF-7 cells was less affected by 300 μM CoCl_2_ treatment than MCF-7 cells transduced with scrambled shRNA, suggesting that restrained growth of MCF-7 cells under CoCl_2_ is linked to the expression of HIF1α (Fig. 4a). The slight reduction in growth rate observed in HIF1α-suppressed MCF-7 at later timepoints could result from incomplete knockdown efficiency, as HIF1α expression is observed in some cells following CoCl_2_ treatment (Additional file 1: Figure S6). In addition, a significantly higher number of Ki67-positive cells was observed in HIF1α-suppressed MCF-7 cells after 4 days of 300 μM CoCl_2_ treatment compared to MCF-7 cells transduced with scrambled shRNA (Fig. 4b). Collectively, these results demonstrate that the hypoxia-mimicking effects of CoCl_2_ are responsible for its ability to modulate cancer dormancy.

### CoCl_2_ treatment induces dormancy in ovarian cancer cells

**Fig. 5:**
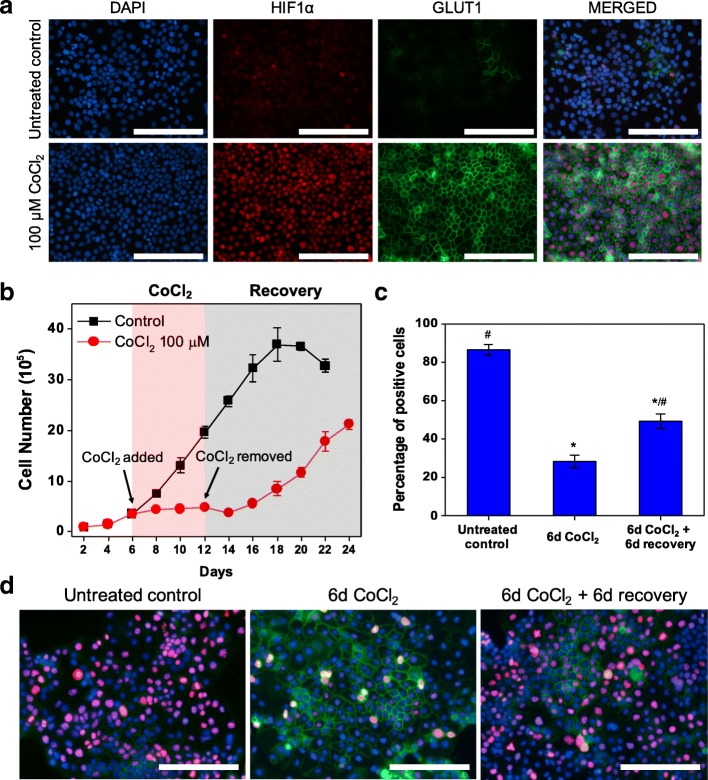
CoCl_2_ treatment also induces dormancy in OVCAR-3 cells. **a** Representative fluorescence images of HIF1α and GLUT1 expression in OVCAR-3 cells after 72 h of 100 μM CoCl_2_ treatment compared to untreated control cells. Scale bars indicate 200 μm. **b** Cell growth analysis of OVCAR-3 cells treated with 100 μM CoCl_2_ for 6 days (from day 6 to day 12) compared to untreated cells. Cells recovered in normal growth media after the 6-day CoCl_2_ treatment. **c** Percentages of Ki67-positive cells in untreated control, 6-day 100 μM CoCl_2_ treatment, and 6-day 100 μM CoCl_2_ treatment followed by 6-day recovery in normal growth media (^*^ *P* < 0.001 compared to untreated control; ^#^ P < 0.001 compared to 6-day CoCl_2_ treatment). Quantification was performed with ImageJ software. **d** Representative fluorescence images of Ki67 (red), GLUT1 (green) and nuclei (blue) in OVCAR-3 cells in untreated control, 6-day 100 μM CoCl_2_ treatment, and 6-day 100 μM CoCl_2_ treatment followed by 6-day recovery in normal growth media. Scale bars indicate 200 μm

To confirm that the link between hypoxic microenvironments and cancer dormancy extends to other types of cancer, we subsequently investigated the ovarian adenocarcinoma cell line OVCAR-3 under CoCl_2_ treatment. OVCAR-3 cells were found to have higher sensitivity to CoCl_2_ than MCF-7 cells, showing similar hypoxic responses at 100 μM CoCl_2_ to the MCF-7 response at 300 μM CoCl_2_ (Fig. 5). OVCAR-3 cells treated with 100 μM CoCl_2_ exhibited significant upregulation in HIF1α and GLUT1 expression compared with untreated cells, indicating that 100 μM CoCl_2_ generated an effective hypoxia-mimicking microenvironment in OVCAR-3 cultures (Fig. 5a). Furthermore, growth patterns similar to MCF-7 under both CoCl_2_ treatment and recovery were observed by cell growth analysis (Fig. 5b). In addition, Ki67 expression was markedly attenuated in cells showing restrained growth during CoCl_2_ treatment (Fig. 5c, d), with 28.4 ± 3.3% Ki67-positive cells in CoCl_2_-treated populations, compared to 86.4 ± 2.8% in untreated populations. Importantly, reversible quiescence of OVCAR-3 cells was also observed by Ki67 expression, with the percentage of Ki67-positive cells increasing from 28.4 ± 3.3% after CoCl_2_ treatment to 49.5 ± 3.7% after 6 days of recovery in normal growth media (Fig. 5c, d). Flow cytometric analysis of PI staining provided further evidence of reversible quiescence, with the percentage of G0/G1 phase cells increasing from 62.6 ± 1.2% in untreated cells to 78.2 ± 0.4% under CoCl_2_ treatment and returning to 62.8 ± 0.7% after 6 days of recovery in normal growth media (Additional file 1: Figure S7). These results show that CoCl_2_ can also be used to induce and maintain dormancy in OVCAR-3 cells by establishing a hypoxia-mimicking microenvironment.

### CoCl_2_-induced hypoxia-mimicking conditions recapitulate heterogeneous cellular response to hypoxia in breast cancer cell lines

**Fig. 6:**
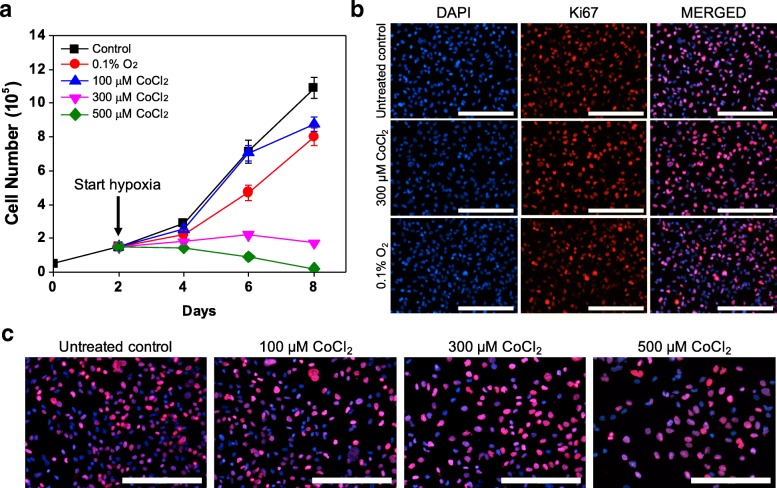
In vitro cellular responses to hypoxia in ER-negative MDA-MB-231 cells can be mimicked by CoCl_2_. **a** Cell growth analysis of MDA-MB-231 cells treated with CoCl_2_ (100, 300 and 500 μM) or true hypoxic conditions (0.1% O_2_) for 6 days (from day 2 to day 8) compared to untreated cells. **b** Representative fluorescence images of Ki67 (red) and nuclei (blue) in MDA-MB-231 cells after 4 days of treatment with 300 μM CoCl_2_ or 0.1% O_2_, compared to untreated control cells (day 2 of culture). **c** Representative fluorescence images of Ki67 (red) and nuclei (blue) in MDA-MB-231 cells at day 6 (after 4-day CoCl_2_ treatment) compared to untreated control cells (day 2 of culture). Scale bars indicate 200 μm

It has been demonstrated that cellular responses to hypoxia vary depending on subtypes of cancer cells [11, 26]. For example, unlike estrogen receptor (ER)-positive MCF-7 cells, hypoxic stress alone is not sufficient to induce dormancy in vitro in ER-negative MDA-MB-231 cells [11]. Given that the in vitro cellular response of ER-positive MCF-7 cells to CoCl_2_ treatment matched that of true hypoxia, we hypothesized that the same would hold for ER-negative breast cancer cells. To evaluate this hypothesis, ER-negative MDA-MB-231 cells were tested with CoCl_2_ and true hypoxic conditions (0.1% O_2_). Growth curve analyses showed that unlike MCF-7 cells, MDA-MB-231 cells did not exhibit restrained cell growth under true hypoxic conditions (0.1% O_2_) (Fig. 6a). Although MDA-MB-231 cell growth was inhibited at higher doses of CoCl_2_, given the lack of reduction in Ki67 expression (Fig. 6b) and the significant cell death observed (Additional file 1: Figure S2), it is likely that the restrained cell growth can be primarily attributed to cell death, not induction of dormancy. Immunofluorescence analysis also showed that cellular responses of MDA-MB-231 to true hypoxia and CoCl_2_ treatment were similar. HIF1α expression was upregulated in both true hypoxia and CoCl_2_ treatment (Additional file 1: Figure S8). In addition, no significant upregulation in GLUT1 was seen (Additional file 1: Figure S8), and neither CoCl_2_ treatment nor exposure to true hypoxia led to a decrease in Ki67 expression (Fig. 6c). These results suggest that CoCl_2_ can recapitulate the in vitro cellular response to true hypoxia in heterogeneous breast cancer cell lines, further demonstrating the robustness of this platform for evaluating induction of breast cancer dormancy under hypoxia-mimicking microenvironments.

### 3D cell culture models combined with CoCl_2_ recapitulate cancer dormancy under hypoxia

**Fig. 7:**
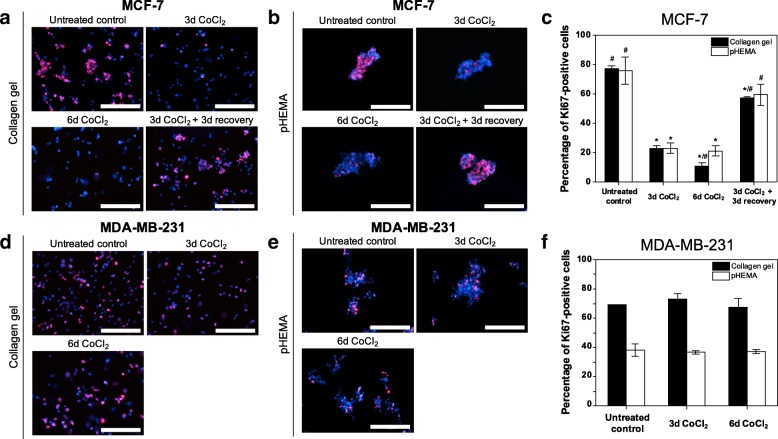
Differential hypoxic regulation of dormancy in MCF-7 and MDA-MB-231 cells can be recapitulated by CoCl_2_ in 3D culture models. **a**-**c** Representative fluorescence images of cycling marker Ki67 (red) and nuclei (blue) in MCF-7 cells embedded in collagen gels (**a**) or grown in pHEMA-coated plates (**b**), and quantification of the percentage of Ki67-positive cells (**c**) in each condition: untreated, 3-day 300 μM CoCl_2_ treatment, and 3-day 300 μM CoCl_2_ treatment followed by 3-day recovery in normal growth media (^*^ P < 0.001 compared to untreated control; ^#^ *P* < 0.005 compared to 3-day CoCl_2_ treatment). **d**-**f** Representative fluorescence images of cycling marker Ki67 (red) and nuclei (blue) in MDA-MB-231 cells embedded in collagen gels (**d**) or grown in pHEMA-coated plates (**e**), and quantification of the percentage of Ki67-positive cells (**f**) in each condition: untreated, 3-day 300 μM CoCl_2_ treatment, and 6-day 300 μM CoCl_2_ treatment. Quantification was performed with ImageJ software. Scale bars indicate 200 μm

We also demonstrated that CoCl_2_ can be combined with 3D cell culture models to investigate cancer dormancy under 3D hypoxic microenvironments. For 3D models, cells were embedded in collagen gels or grown in non-adhesive pHEMA-coated plates. MCF-7 and MDA-MB-231 cells cultured in 3D were found to have stabilized HIF1α expression in response to CoCl_2_ (Additional file 1: Figure S9), but only MCF-7 cells exhibited hallmarks of the dormant state, consistent with our observations in in 2D culture. Ki67 expression in 3D-cultured MCF-7 cells decreased from 75.6 ± 9.3% (pHEMA) or 77.2 ± 1.9% (collagen gel) to 21.3 ± 3.6% (pHEMA) or 23.0 ± 2.0% (collagen gel) after 3-day CoCl_2_ treatment, and recovered to 59.4 ± 7.3% (pHEMA) or 57.2 ± 1.1% (collagen gel) following 3-day recovery in CoCl_2_-free normal growth media (Fig. 7a-c). Unlike MCF-7, MDA-MB-231 cells exhibited no significant change in Ki67 expression, which is also consistent with the results from 2D culture (Fig. 7d-f). Cell cycle analysis also demonstrated reversible quiescence of MCF-7 cells, with an increased percentage of cells arrested in G0/G1 phase under CoCl_2_ treatment compared to untreated cells, and release from G0/G1 phase upon removal of CoCl_2_ in both 3D culture models (Additional file 1: Figure S11). Importantly, these key features of the dormant state were also found in 3D-cultured MCF-7 cells under true hypoxic conditions (0.1% O_2_), suggesting that the 3D CoCl_2_-based platform can also recapitulate cellular responses to hypoxia (Additional file 1: Figure S10 and S11). Furthermore, MCF-7 cells cultured in 2D and 3D systems under CoCl_2_ treatment exhibited similar key signaling features of dormant cells. Western blot analysis of p38 MAPK and ERK(1/2) activity showed that MCF-7 cells treated with CoCl_2_ for 6 days had an increased ratio of p38 MAPK phosphorylation to ERK(1/2) phosophorylation, which has been reported as a signaling hallmark of the dormant state [21, 27], in both 2D and 3D cultures (Additional file 1: Figure S12). These results suggest that the CoCl_2_-based platform is not limited to 2D culture but also can be combined with well-established 3D cell culture models to study cancer dormancy under hypoxic microenvironments.

### CoCl_2_ treatment differentially regulates gene and protein expression in MCF-7 and MDA-MB-231 cells

**Fig. 8:**
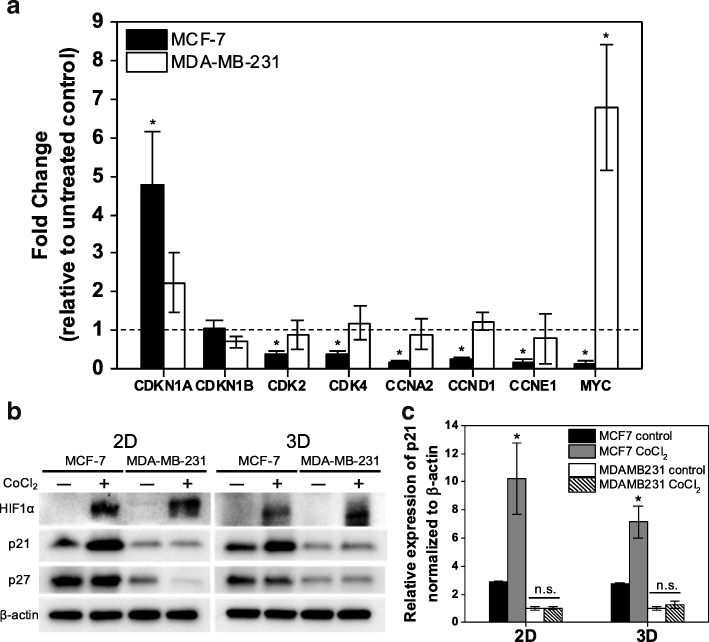
MCF-7 and MDA-MB-231 cells exhibit differential gene and protein expression profiles in response to CoCl_2_ treatment. **a** Fold change in mRNA expression of *CDKN1A*, *CDKN1B, CDK2, CDK4, CCNA2, CCND1, CCNE1,* and *MYC* by qRT-PCR after 72 h of CoCl_2_ treatment relative to untreated control (^*^ *P* < 0.05 compared to untreated control). **b** Western blot analysis of HIF1α, p21, p27 and β-actin (control) expression in MCF-7 and MDA-MB-231 cells after 72 h of CoCl_2_ treatment in 2D and 3D (pHEMA-coated plate) cultures compared to untreated control. **c** Relative protein expression of p21 normalized to β-actin, with results represented as mean ± SD of three independent experiments (^*^ P < 0.05 compared to untreated control)

To investigate possible molecular mechanisms that lead to differential responses to hypoxic microenvironments in MCF-7 and MDA-MB-231 cells, expression profiles of cell cycle-associated genes under CoCl_2_ treatment were examined. Protein and gene expression of the CDK inhibitor p27 (*CDKN1B*) showed no upregulation in response to CoCl_2_ treatment in MCF-7 cells, while p21 (*CDKN1A*) was significantly upregulated by CoCl_2_. In contrast, no significant change in the expression of either p27 or p21 was observed in MDA-MB-231 cells (Fig. 8a-c). p21 inhibits the cell cycle through inactivation of multiple CDK-cyclin complexes [28, 29]: CDK2-cyclin E (*CCNE1*), CDK2-cyclin A (*CCNA2*) and CDK4-cyclin D1 (*CCND1*). Accordingly, p21 upregulation was accompanied by downregulation of *CDK2, CCNE1, CCNA2, CDK4,* and *CCND1* in MCF-7 cells treated with CoCl_2_ (Fig. 8a). Furthermore, *MYC*, a proto-oncogene that plays an integral role in hypoxic adaptation [30], also exhibited differential expression, with significant downregulation in MCF-7 cells and upregulation in MDA-MB-231 cells upon treatment. These findings collectively suggest that CoCl_2_ treatment in MCF-7 and MDA-MB-231 cells impacts different molecular mechanisms, giving rise to their distinct responses to CoCl_2_ treatment.

## Discussion

This study demonstrates that a CoCl_2_-based platform can be used for investigation of cancer dormancy under hypoxia. Several types of models recapitulating the interactions between microenvironments and DTCs have been suggested to date, with a focus on cellular [31] and molecular [32, 33] components such as stromal cells and the extracellular matrix. However, there have been few models focused on non-cellular factors such as oxidative stresses in local microenvironments [34, 35], which may also play a critical role. Previous studies of breast cancer have identified that breast cancer cells under hypoxia became dormant, as evidenced by upregulation of several genes implicated in cancer dormancy [11, 36]. In addition, restrained growth with reversible arrest in G0/G1 phase was observed in breast cancer cells under hypoxia, supporting hypoxic regulation of cancer dormancy [37]. Given the emerging evidence of hypoxic regulation of entrance into the dormant state, the establishment of a robust in vitro model of cancer dormancy under hypoxia is critical for better understanding of mechanisms through which hypoxia induces dormancy of DTCs.

Our results demonstrated that CoCl_2_-induced hypoxia-mimicking conditions can stably trigger and maintain dormancy in MCF-7 cells in a HIF1α-dependent manner. CoCl_2_ treatment showed similar effects to those of a conventional hypoxia chamber in restraining proliferation of MCF-7 cells, reducing expression of cell cycling marker Ki67 in conjunction with upregulation of hypoxia markers HIF1α and GLUT1 and reversible cell cycle arrest in G0/G1 phase. Furthermore, OVCAR-3 ovarian cancer cells also exhibited these hallmarks of dormancy in CoCl_2_-induced hypoxia-mimicking microenvironments. Unlike breast cancer, a direct link between hypoxia and ovarian cancer dormancy has not yet been fully established. However, it has been documented that several pathways including autophagy [38, 39] and p38 signaling [40] that have been implicated in ovarian cancer dormancy are affected by hypoxia. Collectively, these results suggest that this CoCl_2_-based platform can be used as a tool to study dormant cancer cells under hypoxia.

Previous studies have identified multiple effects of CoCl_2_ beyond hypoxia-mimicking effects, such as the development of reactive oxygen species [41] and the activation of NF-κB signaling, which mediates carcinogenesis [42]. Since CoCl_2_ treatment was unable to induce dormancy in MCF-7 cells when HIFα was downregulated in the cells, it is likely that CoCl_2_ is inducing dormancy in a HIF1α-dependent manner. However, given the heterogeneity of cellular responses to hypoxia and CoCl_2_ in different types of cancer, further investigation into alternative effects of CoCl_2_ apart from mimicking hypoxia may be necessary in the future.

Unlike ER-positive MCF-7 cells, ER-negative MDA-MB-231 cells did not exhibit dormant behavior under either true hypoxia or CoCl_2_ treatment. Previous studies have identified heterogeneity in cellular responses to hypoxia in different subtypes of breast cancer cell lines [26, 37, 43]. However, the underlying mechanisms that give rise to their differential ability to enter a dormant state in hypoxic microenvironments have yet to be elucidated. In our study, CDK inhibitor p21 and its associated CDKs and cyclins exhibited contrasting expression in MCF-7 and MDA-MB-231 cells under CoCl_2_ treatment. These results are consistent with previous findings that differential regulation of p21 in MCF-7 and MDA-MB-231 cells in response to various environmental stimuli impacts cell cycle progression [44, 45], and they suggest that the distinct responses to hypoxic stress we observed in these two cell lines are in part p21-mediated. Interestingly, expression of *MYC*, which represses p21 in normoxia [30, 46], exhibits the opposite trend as p21 in MCF-7 and MDA-MB-231 cells. Previous studies have found that hypoxia induces p21 activation by repressing *MYC* through a different regulatory mechanism from the classical hypoxia-inducible genes such as *GLUT1* [30, 46]. Thus, MDA-MB-231 cells may overcome hypoxia-induced cell cycle arrest by overexpressing *MYC*, which in turn deactivates p21 to enable cell cycle progression. Another CDK inhibitor (p27) was not significantly upregulated in either cell line under CoCl_2_ treatment. Given that hypoxia can induce cell cycle arrest independent of p27 or p21 [47], our findings suggest that dormancy observed in MCF-7 cells under CoCl_2_ treatment is in part mediated by p21 upregulation but not p27. p21 can also trigger cellular senescence in a chronic state of G0 cell cycle arrest [29]. However, low activity of senescence-associated β-galactosidase in MCF-7 cells under CoCl_2_ treatment indicated that cells were not in a senescent state. In addition, an increased ratio of p38 MAPK activity to ERK(1/2) activity, which has been associated with p21 activation in dormant tumor cells [27, 48], was observed in MCF-7 cells under CoCl_2_ treatment, providing further evidence of p21-mediated dormancy. Overall, our findings suggest that MCF-7 and MDA-MB-231 employ different molecular mechanisms that are in part regulated by p21-mediated pathways in response to hypoxic microenvironments. Although the role of p21 in hypoxic regulation of dormancy remains to be determined, these findings suggest that our platform can be used to investigate molecular mechanisms underlying hypoxic regulation of cancer dormancy.

As with other in vitro models, this simplified platform also reflects limited aspects of in vivo tumor microenvironments. It lacks geometrical complexity, cellular components including immune cells and organ-specific stromal cells, and extracellular matrix components. However, as opposed to conventional hypoxia models that rely on a hypoxic chamber, the CoCl_2_-based platform can be readily integrated with previously established models centered on cellular and molecular components to better mimic in vivo tumor microenvironments conducive to cancer dormancy. For example, we demonstrated that CoCl_2_ can be combined with 3D cell culture models, which more closely mimic the in vivo tumor microenvironment than 2D monolayer cell culture [48]. Our results showed that CoCl_2_ recapitulated differential hypoxic regulation of cancer dormancy in MCF-7 and MDA-MB-231 cells in two different 3D models, further indicating the robustness of this platform. Furthermore, this platform allows for real-time characterization of dormant cancer cells that has not been practical with hypoxic chambers due to the extremely short half-life of HIF1α (t_1/2_ < 5 min) upon reoxygenation [28]. Thus, oxygen entering the chamber at each opening results in re-oxygenation that can disrupt the hypoxic response [13, 49]. In addition, the ability to generate a large population of dormant cells in the CoCl_2_ platform could enable the investigation of the heterogeneity among dormant cancer cells under hypoxia. Given that hypoxia and cancer dormancy have been associated with limiting the effectiveness of chemotherapy and increasing the risk for recurrence, resulting in poor clinical outcomes [50, 51], information extracted from dormant cancer cells has the potential to identify novel therapeutic strategies for preventing recurrence. Taken together, the CoCl_2_-based platform we have established in this report provides an enabling tool that has potential use in the investigation of undiscovered mechanisms of cancer dormancy regulation under hypoxic microenvironments.

## Conclusion

There have been a limited number of studies on cancer dormancy under hypoxic microenvironments in part due to a lack of well-established platforms. In the present study, we report a facile CoCl_2_-based in vitro platform mimicking hypoxic regulation of cancer dormancy as well as recapitulating differing responses to hypoxia among breast cancer cell lines. A critical advantage of this CoCl_2_-based platform over conventional systems is the ability to stably induce and maintain dormancy in vitro, even in the presence of oxygen. Thus, this platform enables investigation of the poorly-understood molecular mechanisms underlying hypoxic regulation of cancer dormancy, offering a tool to develop potential therapeutic strategies to reduce tumor recurrence.

## Additional file

Additional file 1: **Figure S1.** Similar upregulation of hypoxia markers is observed in MCF-7 cells in response to CoCl_2_ treatment and true hypoxia. **Figure S2.** CoCl_2_ treatment shows differential effects on cell viability in MCF-7 and MDA-MB-231 cells. **Figure S3.** Quantification of Ki67 positive MCF-7 cells upon CoCl_2_ treatment and recovery. **Figure S4.** Cell cycle analysis demonstrates ability of dormant MCF-7 cells to re-enter cell cycle following removal of CoCl_2_. **Figure S5.** Similar β-galactosidase activity levels are observed in CoCl_2_-treated and untreated MCF-7 cells. **Figure S6.** Suppression of HIF1α expression in MCF-7 cells via shRNA. **Figure S7.** Quiescent OVCAR-3 cells exhibit reversible arrest in G0/G1 phase of the cell cycle. **Figure S8.** MDA-MB-231 cells exhibit less upregulation of HIF1α compared to MCF-7 cells and no significant change in GLUT1 expression under CoCl_2_ treatment. **Figure S9.** Similar upregulation of HIF1α is observed in 3D culture models exposed to CoCl_2_ or hypoxia. **Figure S10.** Differential Ki67 expression in response to true hypoxia is observed in MCF-7 and MDA-MB-231 cells in 3-D culture systems. **Figure S11.** Induction of quiescence under hypoxia can be recapitulated by CoCl_2_ in 3D cell culture models. **Figure S12.** CoCl_2_-treated MCF-7 cells exhibit an increased p38 to ERK activity ratio, a signaling hallmark of dormant state, in both 2D and 3D models. (DOCX 12288 kb)

## Abbreviations

CDK: Cyclin dependent kinase
DTCs: Disseminated tumor cells
ER: Estrogen receptor
FBS: Fetal bovine serum
GLUT1: Glucose transporter 1
HIF1α: Hypoxia inducible factor 1-alpha
PI: Propidium iodide
PS: Penicillin-streptomycin
shRNA: Short hairpin RNA
VHL: von Hippel Lindau
