# Supplementary material for: A facile in vitro platform to study cancer cell dormancy under hypoxic microenvironments using CoCl2
Source: J Biol Eng. 2018 Aug 3;12:12. doi: 10.1186/s13036-018-0106-7 (PMC6091074; doi:10.1186/s13036-018-0106-7)
Supplement: Supplementary file 1 — Figure S1. Similar upregulation of hypoxia markers is observed in MCF-7 cells in response to CoCl2 treatment and true hypoxia. Figure S2. CoCl2 treatment shows differential effects on cell viability in MCF-7 and MDA-MB-231 cells. Figure S3. Quantification of Ki67 positive MCF-7 cells upon CoCl2 treatment and recovery. Figure S4. Cell cycle analysis demonstrates ability of dormant MCF-7 cells to re-enter cell cycle following removal of CoCl2. Figure S5. Similar β-galactosidase activity levels are observed in CoCl2-treated and untreated MCF-7 cells. Figure S6. Suppression of HIF1α expression in MCF-7 cells via shRNA. Figure S7. Quiescent OVCAR-3 cells exhibit reversible arrest in G0/G1 phase of the cell cycle. Figure S8. MDA-MB-231 cells exhibit less upregulation of HIF1α compared to MCF-7 cells and no significant change in GLUT1 expression under CoCl2 treatment. Figure S9. Similar upregulation of HIF1α is observed in 3D culture models exposed to CoCl2 or hypoxia. Figure S10. Differential Ki67 expression in response to true hypoxia is observed in MCF-7 and MDA-MB-231 cells in 3-D culture systems. Figure S11. Induction of quiescence under hypoxia can be recapitulated by CoCl2 in 3D cell culture models. Figure S12. CoCl2-treated MCF-7 cells exhibit an increased p38 to ERK activity ratio, a signaling hallmark of dormant state, in both 2D and 3D models. (DOCX 12288 kb) [file 13036_2018_106_MOESM1_ESM.docx]

**Supplementary Information**

**A facile *in vitro* platform to study cancer cell dormancy under hypoxic microenvironments using CoCl_2_**

Hak Rae Lee, Faith Leslie, Samira M. Azarin

**Supplementary Materials:**

Figures S1 - S12

**Figure S1. Similar upregulation of hypoxia markers is observed in MCF-7 cells in response to CoCl_2_** **treatment and true hypoxia.** (A,B) Western blot analysis of HIF1α and β-actin (control) expression in MCF-7 cells to evaluate effects of duration and dose of CoCl_2_ treatment (A) and to compare 72 hour treatment with 300 µM CoCl_2_ or true hypoxia (0.1% O_2_) (B). In (B), lanes 1-4 represent untreated control (lane 1), MCF-7 cells treated with 300 µM CoCl_2_ (lane 2), HIF1α-silenced MCF-7 cells treated with 300 µM CoCl_2_ (lane 3), and MCF-7 cells treated with true hypoxia (0.1% O_2_, lane 4). (C) Representative fluorescence images of HIF1α and GLUT1 expression in MCF-7 cells after 72 hours of treatment with 300 µM CoCl_2_ or 0.1% O_2_ compared to untreated control cells. Nuclei were stained with DAPI. Scale bars indicate 200 µm.


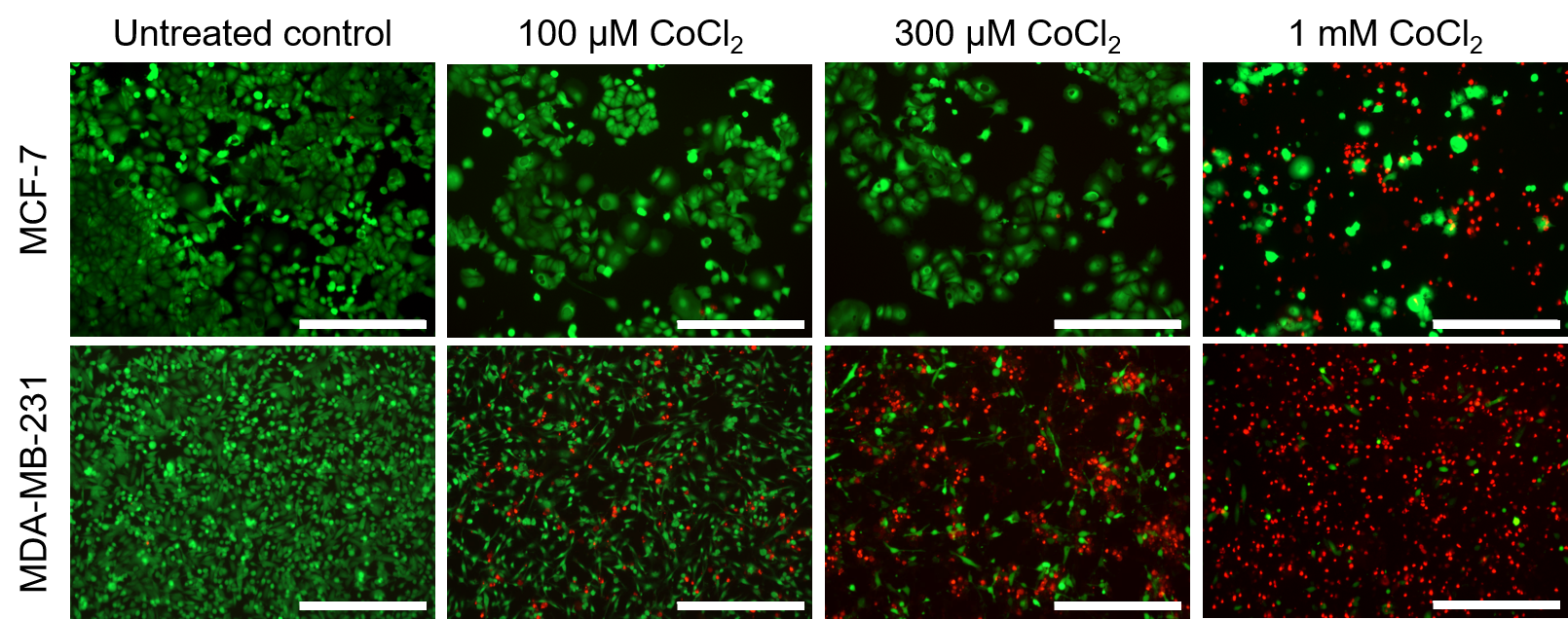


**Figure S2. CoCl_2_ treatment shows differential effects on cell viability in MCF-7 and MDA-MB-231 cells.** Cell viability after 72 hours of treatment with varying concentrations of CoCl_2_ was evaluated with a two-color fluorescence assay in which live cells were stained with calcein AM (green) and dead cells were stained with ethidium homodimer-1 (red). Scale bars indicate 400 µm.


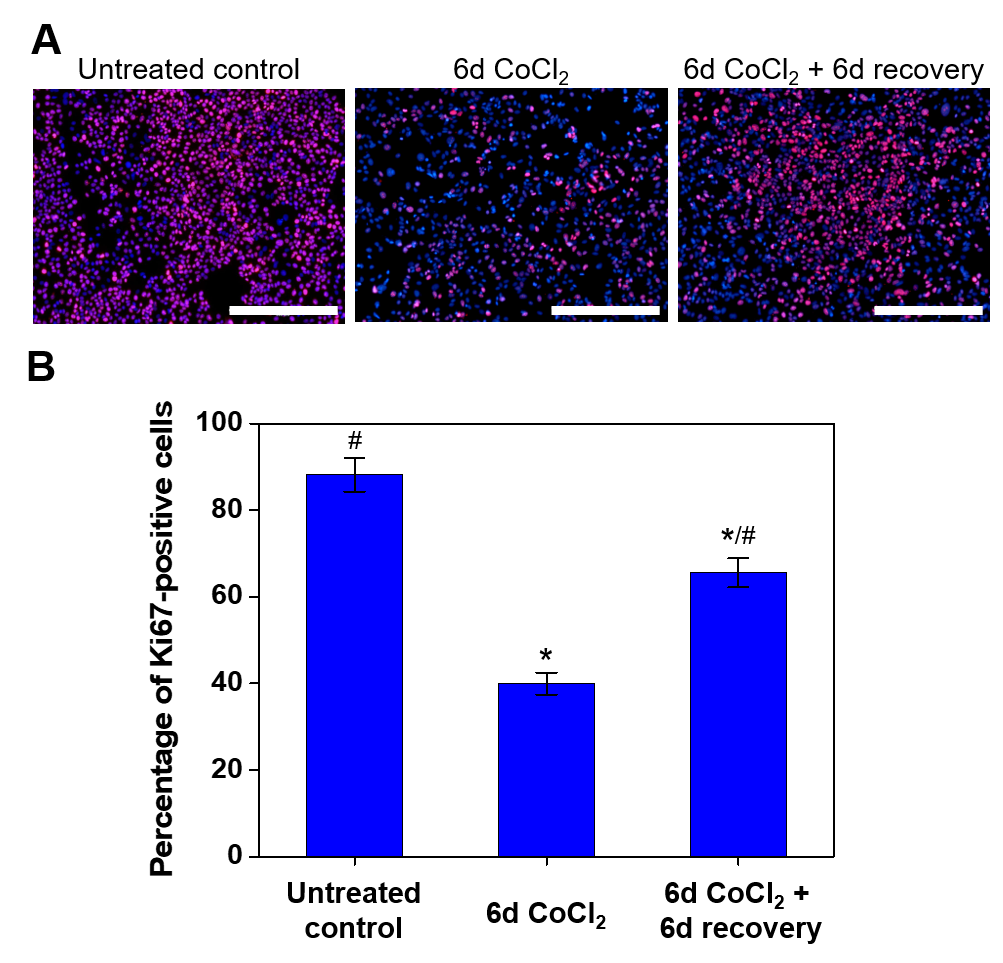


**Figure S3.** **Quantification of Ki67 positive MCF-7 cells upon CoCl_2_ treatment and recovery.** (A,B) Representative fluorescence images of Ki67 expression (red) and nuclei (blue) in MCF-7 cells (A) and percentages of Ki67 positive MCF-7 cells (B) in each condition: untreated, 6-day 300 µM CoCl_2_ treatment, and 6-day 300 µM CoCl_2_ treatment followed by 6-day recovery in normal growth media (^*^ P < 0.001 compared to untreated control; ^#^ P < 0.001 compared to 6-day CoCl_2_ treatment). Quantification was performed with ImageJ software. Scale bars indicate 400 µm.


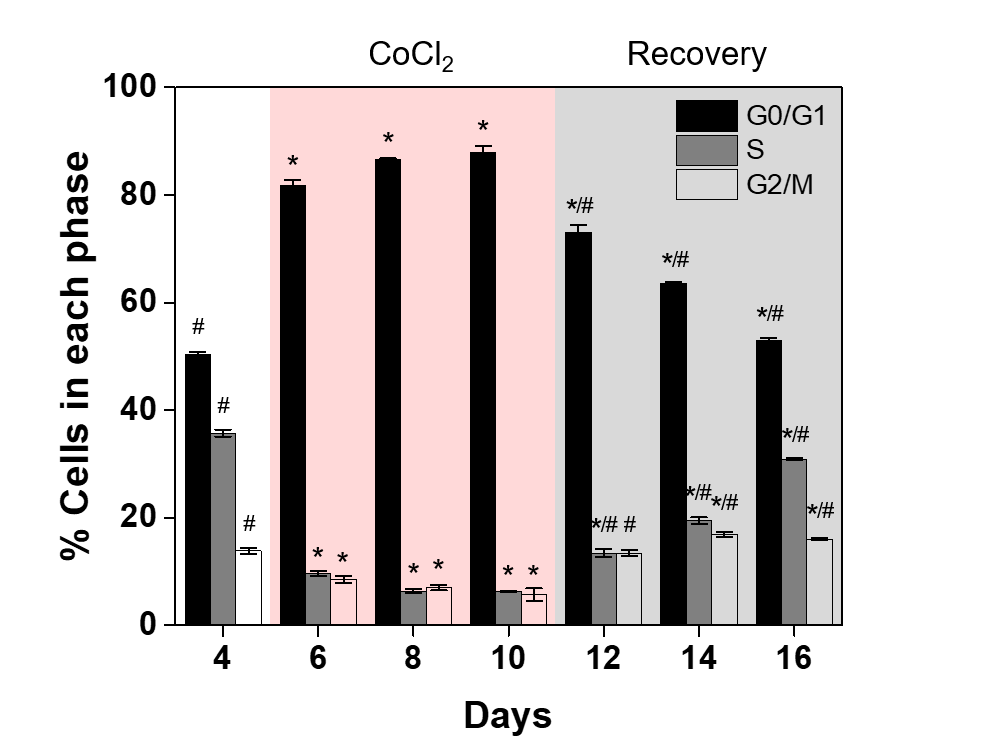


**Figure S4.** **Cell cycle analysis demonstrates ability of dormant MCF-7 cells to re-enter cell cycle following removal of CoCl_2_.** Flow cytometric analysis of PI staining intensity in MCF-7 cells treated with 300 µM CoCl_2_ for 6 days (from day 4 to day 10), followed by recovery (from day 10 to day 16), compared to untreated control at day 4. Cell populations are reported as percentage of cells in each phase. Data were analyzed by Modfit LT software (^*^ P < 0.01 compared to untreated control (day 4); ^#^ P < 0.01 compared to 6-day CoCl_2_ treatment (day 10)).

**Figure S5.** **Similar β-galactosidase activity levels are observed in CoCl_2_-treated and untreated MCF-7 cells.** Representative bright field images of MCF-7 cells stained with β-galactosidase in each condition: untreated, 6-day 300 µM CoCl_2_ treatment followed by 2-day recovery in normal growth media, and 6-day 12.5 µM etoposide treatment followed by 2-day recovery in normal growth media (positive control).


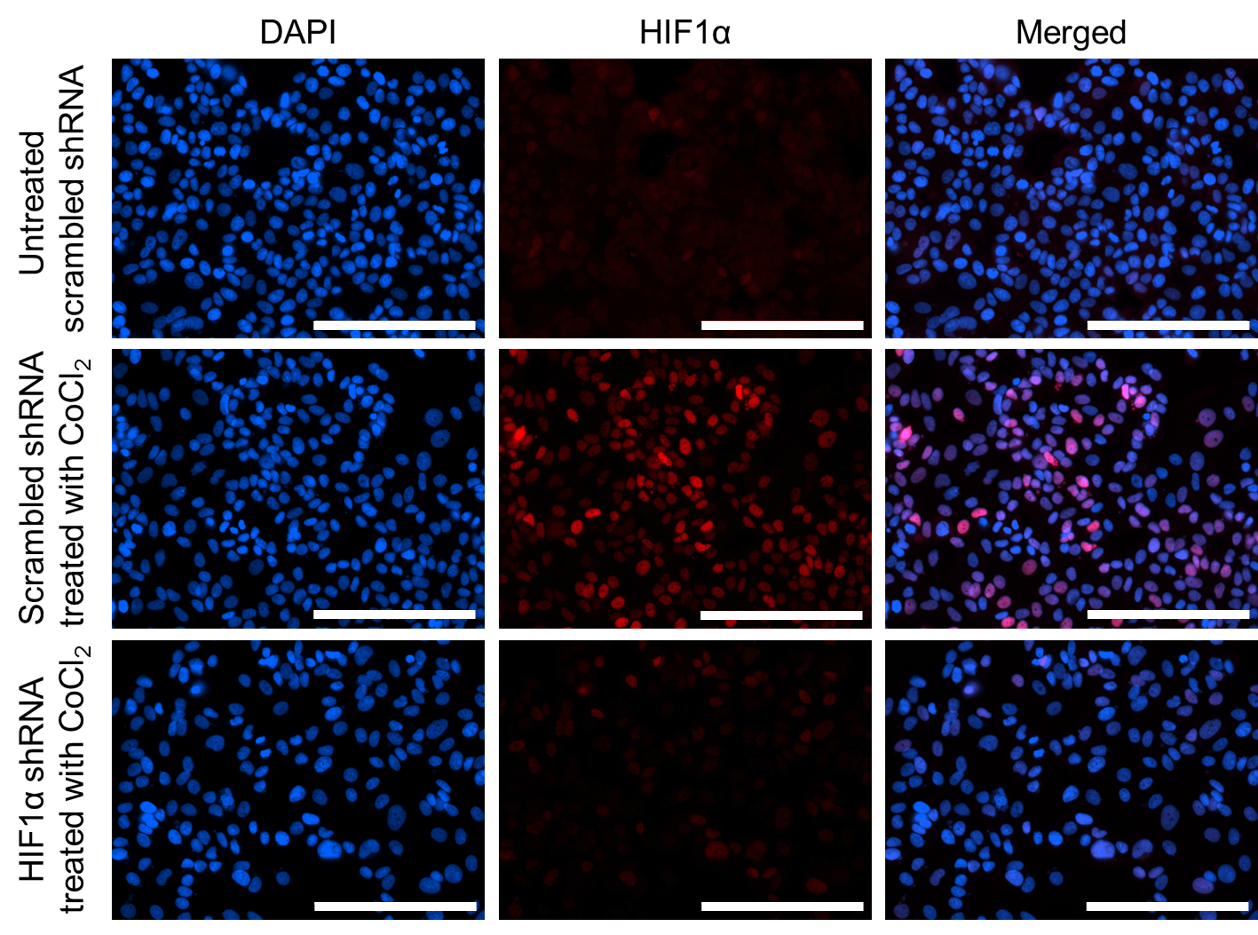


**Figure S6. Suppression of HIF1α expression in MCF-7 cells via shRNA.** Representative fluorescence images of HIF1α expression in MCF-7 cells transduced with HIFα-specific shRNA (bottom row) and scrambled shRNA (middle row) after 72 hours of treatment with 300 µM CoCl_2_ compared to untreated MCF-7 cells transduced with scrambled shRNA (top row). Nuclei were stained with DAPI. Scale bars indicate 200 µm.


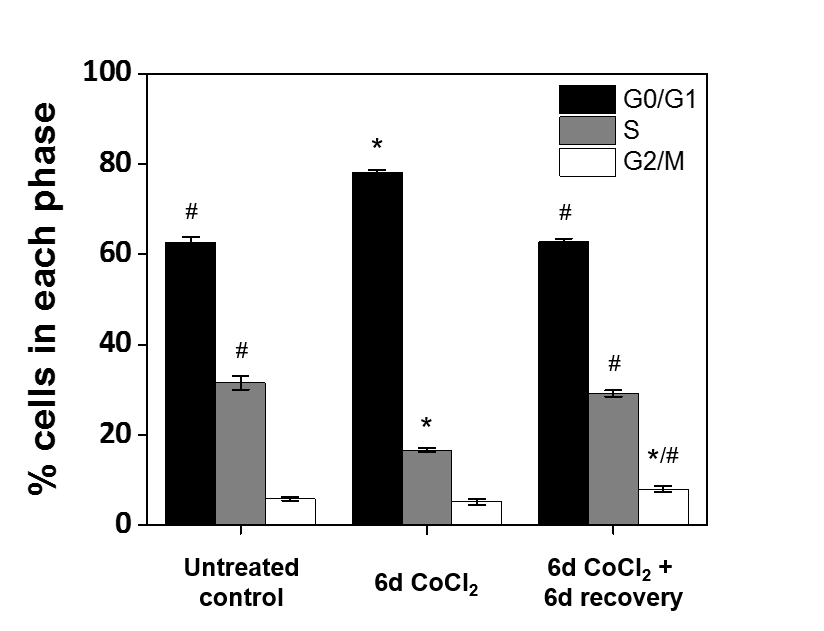


**Figure S7.** **Quiescent OVCAR-3 cells exhibit reversible arrest in G0/G1 phase of the cell cycle.** Flow cytometric analysis of OVCAR-3 cell cycle distribution using PI following 6-day treatment with 100 µM CoCl_2_, or 6-day treatment with 100 µM CoCl_2_ followed by 6-day recovery in normal growth media, compared to untreated control. Cell populations are reported as percentage of cells in each phase. Data were analyzed by Modfit LT software (^*^ P < 0.05 compared to untreated control; ^#^ P < 0.05 compared to 6-day CoCl_2_ treatment).

**
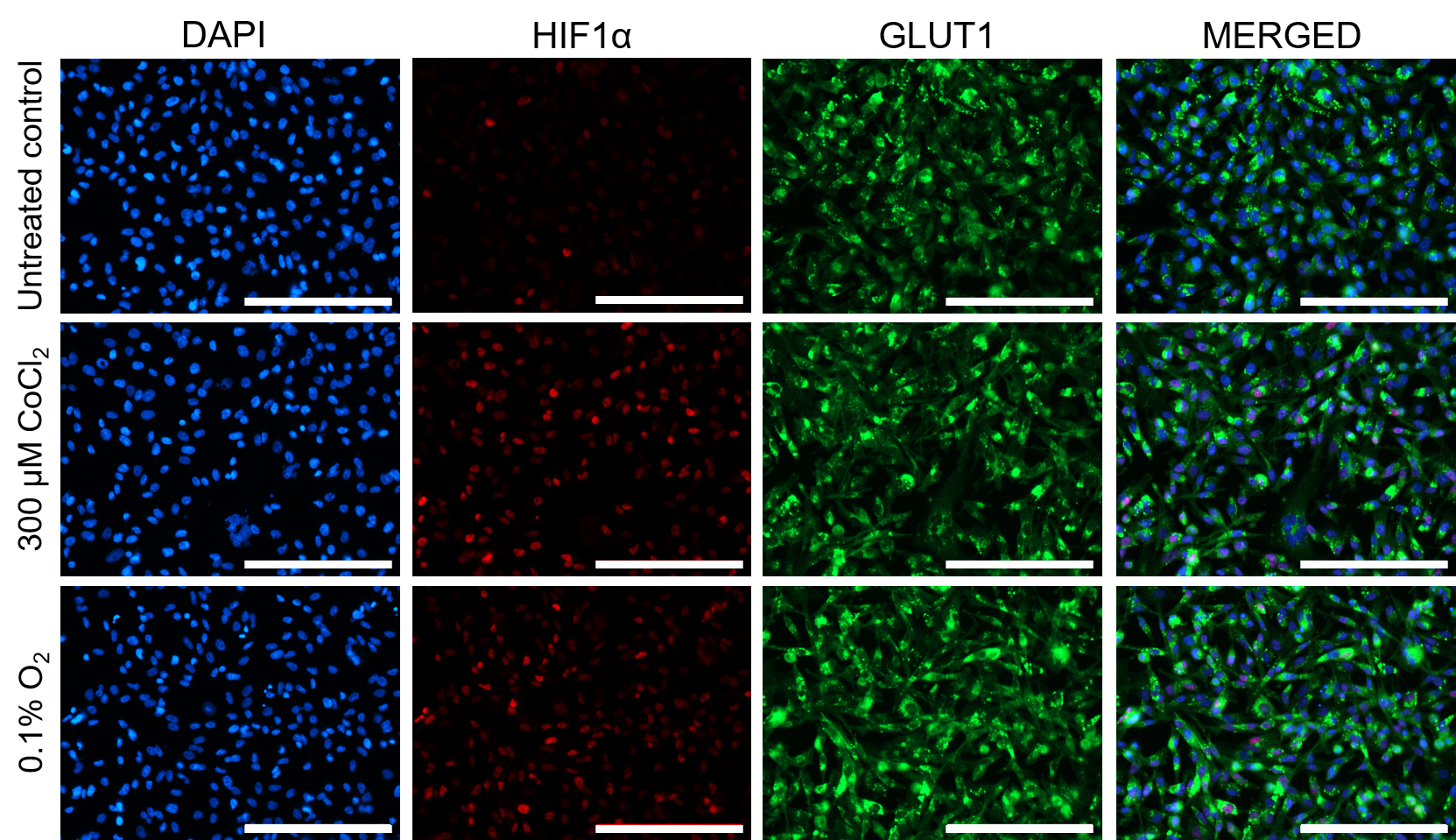
**

**Figure S8. MDA-MB-231 cells exhibit less upregulation of HIF1α compared to MCF-7 cells and no significant change in GLUT1 expression under CoCl_2_ treatment.** Representative fluorescence images of HIF1α and GLUT1 expression in MDA-MB-231 cells after 72 hours of treatment with 300 µM CoCl_2_ or 0.1% O_2_ compared to untreated control cells. Nuclei were stained with DAPI. Scale bars indicate 200 µm.

**Figure S9. Similar upregulation of HIF1α** **is observed in 3D culture models exposed to CoCl_2_ or hypoxia.** Representative fluorescence images of HIF1α expression in MCF-7 and MDA-MB-231 cells embedded in collagen gels (A) or grown in pHEMA-coated plates (B) after 3 days of treatment with 300 µM CoCl_2_ or 0.1% O_2_ compared to untreated control cells. Nuclei were stained with DAPI. Scale bars indicate 200 µm.

**Figure S10. Differential Ki67 expression in response to true hypoxia is observed in MCF-7 and MDA-MB-231 cells in 3D culture systems.** Representative fluorescence images of Ki67 expression in MCF-7 and MDA-MB-231 cells embedded in collagen gels (A) or grown in pHEMA-coated plates (B) in each condition: untreated, 3-day treatment with 0.1% O_2_, and 3-day treatment with 0.1% O_2_ followed by 3-day recovery in normal growth media (MCF-7 only). Nuclei were stained with DAPI. Scale bars indicate 200 µm.

**Figure S11. Induction of quiescence under hypoxia can be recapitulated by CoCl_2_ in 3D cell culture models.** (A,B) Flow cytometric analysis of PI staining in MCF-7 cells embedded in collagen gels (A) or grown in pHEMA-coated plates (B). Cells were exposed to 300 µM CoCl_2_ treatment (left) or true hypoxic conditions (0.1% O_2_, right). Cell populations are reported as percentage of cells in each phase. Data were analyzed by Modfit LT software (^*^ P < 0.05 compared to untreated control; ^#^ P < 0.05 compared to 3-day treatment with the respective condition).

**Figure S12. CoCl_2_-treated MCF-7 cells exhibit an increased p38 to ERK activity ratio, a signaling hallmark of dormant state, in both 2D and 3D models.** (A) Western blot analysis of p38 MAPK, phosphorylated p38 (pp38) MAPK, ERK(1/2) and phosphorylated ERK(1/2) (pERK(1/2)) in MCF-7 cells after 72 hours of CoCl_2_ treatment compared to untreated control in 2D and 3D (pHEMA-coated plate) cultures. (B) Quantification of p38 to ERK signaling activity ratios in 2D and 3D-cultured MCF-7 cells from the western blot analysis, with results represented as mean ± SD of three independent experiments (^*^ P < 0.001 compared to untreated control).
